# Comprehensive analysis identified a reduction in ATP1A2 mediated by ARID3A in abdominal aortic aneurysm

**DOI:** 10.1111/jcmm.17301

**Published:** 2022-04-19

**Authors:** Qunhui Wang, Na Li, Xian Guo, Bo Huo, Rui Li, Xin Feng, Zemin Fang, Xue‐Hai Zhu, Yixiang Wang, Xin Yi, Xiang Wei, Ding‐Sheng Jiang

**Affiliations:** ^1^ Division of Cardiothoracic and Vascular Surgery Tongji Hospital Tongji Medical College Huazhong University of Science and Technology Wuhan Hubei China; ^2^ Key Laboratory of Organ Transplantation Ministry of Education NHC Key Laboratory of Organ Transplantation Key Laboratory of Organ Transplantation Chinese Academy of Medical Sciences Wuhan Hubei China; ^3^ Clinical medical College Wuhan University of Science and Technology Wuhan Hubei China; ^4^ 117921 Department of Cardiology Renmin Hospital of Wuhan University Wuhan Hubei China

**Keywords:** abdominal aortic aneurysm, ARID3A, ATP1A2, blood pressure, VSMCs

## Abstract

Abdominal aortic aneurysm (AAA) is characterized by abdominal aorta dilatation and progressive structural impairment and is usually an asymptomatic and potentially lethal disease with a risk of rupture. To investigate the underlying mechanisms of AAA initiation and progression, seven AAA datasets related to human and mice were downloaded from the GEO database and reanalysed in the present study. After comprehensive bioinformatics analysis, we identified the enriched pathways associated with inflammation responses, vascular smooth muscle cell (VSMC) phenotype switching and cytokine secretion in AAA. Most importantly, we identified ATPase Na^+^/K^+^ transporting subunit alpha 2 (ATP1A2) as a key gene that was significantly decreased in AAA samples of both human and mice; meanwhile, its reduction mainly occurred in VSMCs of the aorta; this finding was validated by immunostaining and Western blot in human and mouse AAA samples. Furthermore, we explored the potential upstream transcription factors (TFs) that regulate ATP1A2 expression. We found that the TF AT‐rich interaction domain 3A (ARID3A) bound the promoter of ATP1A2 to suppress its expression. Our present study identified the ARID3A‐ATP1A2 axis as a novel pathway in the pathological processes of AAA, further elucidating the molecular mechanism of AAA and providing potential therapeutic targets for AAA.

## 1 INTRODUCTION

Abdominal aortic aneurysm (AAA) refers to a pathological expansion of the abdominal aorta with diameter >3 cm or 1.5 times the size of the normal aorta, which is characterized by progressive structural impairment and potentially lethal condition with a risk of dissection and rupture.^1^, ^2^, ^3^ Various risk factors associated with AAA formation and progression have been identified, including smoking, hypertension, older age, male sex, hypercholesterolemia, diabetes and genetic conditions.^4^, ^5^ Patients with AAA have no specific symptoms, at most with back, abdominal, groin or testicular pain. Therefore, the rupture of AAA has no preceding symptoms, which leads to a high mortality (approximately 80%) if not treated immediately.^6^, ^7^ However, at present, surgery is the only way to prevent AAA rupture with certain limitations (high cost, high requests for the surgeon and mortality 1–5%), especially for patients with large AAAs.^8^ Therefore, it is necessary to fully understand the molecular and cellular mechanisms underlying AAA initiation and progression to establish an effective therapeutic strategy for AAA patients.

Further research has elucidated the underlying pathophysiological processes responsible for AAA, such as endothelial dysfunction, vascular smooth muscle cell (VSMC) apoptosis and depletion, elastic media destruction, and proteolytic degradation of the extracellular matrix (ECM) proteins elastin and collagen.^5^ The molecular mechanisms that induce these pathological processes have been more or less clarified.^9^, ^10^ For example, infiltration of various immune cells has been regarded as a key feature and driver of AAA, especially macrophages which contribute to the destruction of the aortic normal lamellar architecture.^11^, ^12^ Although immune and inflammation have been proved to be the main culprits in initiation and progression of AAA, there are still lots of questions about the research on the mechanism of inflammatory responses. The most concern is the failure of clinical transformation application based on these findings about immune and inflammation,^8^ which suggests that our understanding of the pathogenesis of AAA is still incomplete. Thus, except immune‐related pathways, exploring the non‐immune molecules involved in AAA formation could provide new insights into the therapeutic strategies of AAA.

**FIGURE 1:**
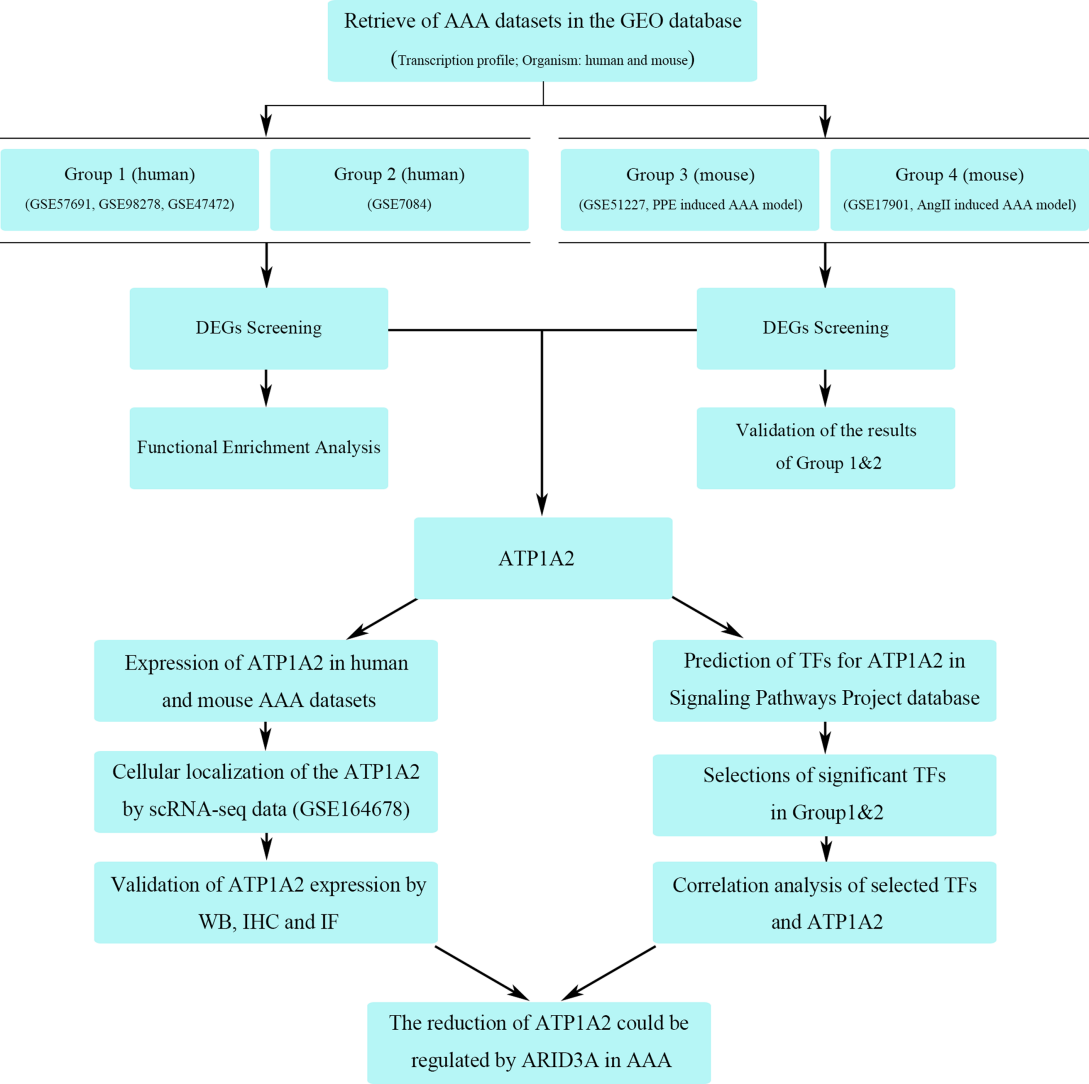
Study flowchart. DEGs: differentially expressed genes; AAA: abdominal aortic aneurysm; PPE: porcine pancreatic elastase; TFs: transcription factors; scRNA‐seq: single‐cell RNA sequencing; WB: Western blot; IHC: immunohistochemical staining; and IF: immunofluorescence staining

In the present study, we reanalysed seven AAA datasets downloaded from the Gene Expression Omnibus (GEO) database (Figure 1). We identified ATP1A2 as the key gene; its expression level was decreased in AAA samples compared with control samples, which was validated by immunostaining and Western blot in human AAA samples and an angiotensin II (Ang II)‐induced mouse model of AAA. The ATP1A2 gene encodes α2‐subunit of Na^+^/K^+^‐ATPase, which is a transmembrane protein responsible for intracellular ion homeostasis critical for membrane potential and numerous cellular processes.^13^, ^14^ It has been reported that a 50% reduction in the α2‐subunit significantly enhances cardiac and vasculature contraction, resulting in an abnormal increase in blood pressure.^13^ Although hypertension is one of the most important risk factors for AAA and ATP1A2 is involved in the regulation of blood pressure, whether ATP1A2 participates in the pathological processes of AAA remains unknown. Therefore, we next performed the single‐cell RNA sequencing (scRNA‐seq) data analysis and screened the transcription factors (TFs) that potentially regulate the expression of ATP1A2, to reveal the underlying mechanisms of ATP1A2 in AAA. Among the four isoforms of the α‐subunit, ATP1A2 was significantly decreased in AAA, and its reduction mainly occurred in VSMCs of the aorta. Furthermore, we found that the transcription factor ARID3A bound the promoter of ATP1A2 to suppress its expression. Thus, our present study identified the ARID3A‐ATP1A2 axis as a novel pathway involved in AAA regulation, which not only provides new insights into the pathogenesis of AAA but also promotes the development of AAA diagnostic and treatment approaches.

## 2 MATERIALS AND METHODS

### 2.1 Human aortic samples acquisition

Full‐thickness aortic samples were obtained from patients (10 AAA, 5 thoracic aortic aneurysm (TAA) and 5 aortic dissection (AD) patients) undergoing AAA or TAA or AD repair operations at Tongji Hospital, Tongji Medical College, Huazhong University of Science and Technology. Normal aortic samples were obtained during heart transplantation as controls. Specimens were collected and stored at −80°C or in 4% paraformaldehyde until assayed. The utilization of human vascular tissues was approved by the ethics committee of Tongji Hospital, Tongji Medical College, Huazhong University of Science and Technology, and the protocol conformed to the ethical guidelines of the Declaration of Helsinki. All patients gave written informed consent.

### 2.2 AAA mouse model

The AAA mouse model was established with Ang II infusion as previously reported.^15^, ^16^ In brief, Alzet osmotic pumps (model 2004; 0000298, Durect Corporation) containing either Ang II (AAA group, 1 μg/kg/min, Sigma‐Aldrich) or saline were implanted in 10‐week‐old ApoE^−/−^ male mice (C57BL/6J background) for 28 days. The saline‐injected mice were assigned to the Sham group. After sacrifice, aneurysmal (proximal to the renal arteries) or control segments of the aorta were harvested for further processing. All experimental protocols for mice in this study were approved by the Animal Experimental Ethics Committee of the Tongji Hospital, Tongji Medical College, Huazhong University of Science and Technology.

### 2.3 Microarray datasets

Datasets search in GEO database (http://www.ncbi.nlm.nih.gov/geo/) using the following terms: ‘Abdominal Aortic Aneurysm or AAA’ retrieved 121 datasets, including 36 human sample datasets and 39 mouse sample datasets in Dec 2020. Then, we screened the datasets according to the organism of datasets (‘human and mouse’), type of sequencing (‘transcription profile’), type of sampling (aortic tissue) and time point of sampling (7 days after AAA in the mouse model). Finally, we downloaded six datasets from the GEO database, which were divided into four groups according to the platforms and species in the present study. The more detailed information about the datasets is shown in Table 1.^16^, ^17^, ^18^, ^19^, ^20^, ^21^

**TABLE 1:** Details of the datasets included in the present study

Groups	Datasets	Platforms	Control	AAA	Species	References
Group 1	GSE98278	GPL10558	0	48	human	^19^
	GSE57691	GPL10558	10	49	human	^18^
	GSE47472	GPL10558	8	14	human	^17^
Group 2	GSE7084	GPL2507	7	6	human	^20^
Group 3	GSE51227	GPL13913	5	5	mouse	^16^
Group 4	GSE17901	GPL4134	6	4	mouse	^21^
scRNA‐seq data	GSE164678	GPL24247	1	1	mouse	^25^

### 2.4 Data analysis

Data preprocessing, including background correction and quartile normalization, was performed by Limma package^22^ for Groups 1&2 and marray package for Group 3&4 in R version 4.1.1. In particular, Group 1 contained three datasets with the same platform, which were merged after removing batch effects from nonbiological technical biases by applying ComBat algorithm of the SVA package.^23^ Limma package was used to screen the differentially expressed genes (DEGs) between the AAA and control groups by setting significance cutoff criteria to adjusted *p*‐value <0.05 corrected by Benjamini–Hochberg multiple test and fold changes (FC) >1.5. Volcano plots and heatmap plots were utilized to display the DEGs between AAA samples and controls by using some of the packages (ggplot2, pheatmap and circlize) provided by the bioconductor project.

### 2.5 Functional enrichment

The clusterProfiler package was applied to carry out Gene Ontology (GO), Kyoto Encyclopedia of Genes and Genomes (KEGG) and Gene Set Enrichment Analysis (GSEA) analysis.^24^ Significantly enriched GO and KEGG terms associated with DEGs were screened out with the threshold *p*‐value <0.05. In addition, GSEA was performed based on the preprocessed data, and gene sets related to KEGG pathway (c2.cp.kegg.v7.5.symbols.gmt) in MSigDB (http://software.broadinstitute.org/gsea/msigdb) served as reference gene sets. The pathways with adjusted *p*‐value < 0.05 were considered to be significantly enriched.

### 2.6 Single‐cell RNA sequencing data analysis

To locate and verify the ATP1A2 expression, we downloaded a scRNA‐seq dataset (GSE164678) of CaCl_2_‐induced AAA mouse model from GEO database.^25^ The scRNA‐seq data filtered with the following criteria: genes detected in >3 cells, cells with >200 distinct genes and percentage of mitochondrial genes <10%. After integrating the AAA and sham data by CAA algorithm of Seurat package (version 4.0.2),^26^ data normalization and scaling were performed and then subjected to dimension reduction at 3 stages of analysis including the selection of variable genes, principal component analysis (PCA), and uniform manifold approximation and projection (UMAP). Cell clustering was assessed across a range of predetermined resolution scales to ensure separation of known major aortic cell types without excessively subclustering (resolution = 0.5). Cell types were then identified by their expression levels of cell‐specific markers according to the CellMarker database.^27^ Next, expressions of ATP1A1‐4 in each cell population were assessed.

### 2.7 Identification of transcription factors (TFs)

To explore whether transcription factors are involved in the dysregulation of ATP1A2, we used web databases to predict TFs for ATP1A2. We selected ‘human’ for the biosample category with other default options in The Signaling Pathways Project (http://www.signalingpathways.org/), which contains manually curated transcriptomic and cistromic (ChIP‐Seq) datasets involving genetic and small molecule manipulations of cellular receptors, enzymes and transcription factors.^28^ After removing redundant TFs, a total of 103 TFs targeting ATP1A2 were identified. Then, we compared the expression of these TFs between the AAA and control samples in Groups 1&2 to screen out the differentially expressed TFs with *p*‐values < 0.05 corrected by Benjamini–Hochberg multiple test.

### 2.8 Correlation analysis

In order to identify the potential TFs that could regulate the ATP1A2 expression, we first screened for the common differentially expressed TFs targeting ATP1A2 in Groups 1&2, and then adopted Pearson correlation analysis to evaluated the correlation between candidate TFs (AR, ARID3A, MXI1 and PGR) and ATP1A2 expression by GGally package (version 2.1.2) in R. Absolute values of Pearson correlation coefficients >0.5 and *p*‐value < 0.05 were used as cut‐off values.

### 2.9 HE and EVG staining

Five‐micrometre paraffin‐embedded sections of mouse abdominal aortic sections were used for haematoxylin–eosin (H&E, G1004&G1002, Servicebio, Wuhan, China) staining and Verhoeff‐Van Gieson (G1042, Servicebio) staining according to the manufacturer's instructions as previously reported.^29^

### 2.10 Western blot

Western blot was performed by the protocol described in our previous studies.^29^, ^30^ The main primary antibodies used in the present study were as follows: anti‐ATP1A1 (1:500, rabbit, 14418–1‐AP, Proteintech), anti‐ATP1A2 (1:500, rabbit, 16836–1‐AP, Proteintech), anti‐Flag (1:1000, mouse, F1804, Sigma) and anti‐β‐actin (1:5000, rabbit, AC026, Abclonal). Mean band intensity was quantified by ImageJ (version 1.53a) and normalized to β‐actin as indicated.

### 2.11 Immunohistochemical (IHC) and immunofluorescence (IF) staining

IHC staining was performed on paraffin‐embedded sections (5 µm) of the AAA/TAA/AD tissues as previously described.^11^ Briefly, sections were incubated overnight with primary antibodies targeting ATP1A1 and ATP1A2 (1:1000, rabbit, 14418–1‐AP/16836‐1‐AP, Proteintech), followed by enhancer solution and biotinylated anti‐IgG and streptavidin‐peroxidase for 30 min at 37°C. Development of the chromogenic colour reaction was accomplished using the peroxidase substrate 3,30‐diamminobenzidine (Maixin, Fuzhou, China) after sections were washed with phosphate‐buffered saline (PBS) three times. IF staining was performed on paraffin‐embedded sections (5 μm) of AAA tissues. Sections were incubated overnight with primary antibodies against ATP1A1/ATP1A2 (1:100, rabbit, 14418–1‐AP/16836‐1‐AP, Proteintech) and α‐SMA (1:200, mouse, ab7817, Abcam), followed by donkey anti‐rabbit Alexa Fluor 568 (A10042, Invitrogen) and donkey anti‐mouse Alexa Fluor 488 (A21202, Invitrogen) secondary antibodies after the sections were washed three times with PBS. Nuclei were counterstained with 4′, 6‐diamidino‐2‐phenylindole (DAPI, BL105A, Biosharp). Images were captured using an Olympus BX51 TRF Fluorescent/light microscope system (Olympus, Tokyo, Japan).

### 2.12 Generation of reporter and expression vectors

The promoter region between −1002 and 0 of the human ATP1A2 gene was amplified by PCR and then inserted into the PGL3 reporter vector between the *XhoI* and *HindIII* sites. The full‐length human ARID3A coding sequence was amplified and cloned into the pHAGE‐CMV expression vector between the *MluI* and *XhoI* restriction sites. The pCDNA3.1‐AR (P8889) expression vector was purchased from Wuhan Miaoling Bio Corporation. The primers used for ATP1A2 promoter amplification were as follows: forward, 5′‐TGCTAGCCCGGGCTCGAGTGTTCTCCAGCCTCAGTCCT‐3′; reverse, 5′‐TACCGGAATGCCAAGCTTGAGAAAGCCAAAGCAACAGG‐3′. The primers used for ARID3A coding region amplification were as follows: forward, 5′‐GAAGACACCGGCGGCCACGCGTGCCACCATGAAACTACAGGCCGTGAT‐3′; reverse, 5′‐GGGCCCTCTAGACTCGAGAGGCAACGAGTTATTTGAGG‐3′.

### 2.13 Luciferase reporter assay

The ability of AR and ARID3A to regulate ATP1A2 expression was measured by dual‐luciferase assay. Briefly, 3 × 10^4^ HEK293 cells were seeded in 48‐well plates, and after 12 hours, the empty vector or AR/ARID3A expression vectors (0.15 μg per well) were transiently cotransfected into cells with ATP1A2‐PGL3 (0.05 μg per well) and TK (0.02 μg per well). ATP1A2‐PGL3 was labelled with firefly luciferase, while TK was labelled with Renilla luciferase. Thirty‐six hours later, cell lysates were harvested, and the firefly and Renilla luciferase activities were detected using a dual‐luciferase reporter assay kit (Promega, E1910). The activity of ATP1A2 luciferase was normalized to that of the empty vector as a control group.

### 2.14 Statistical analysis

Bioinformatics analysis was described above. The data from all experiments are described as the mean ±standard deviation (SD). Two‐tailed Student's *t*‐test was used to identify differences between two groups and one‐way ANOVA followed by Tukey's *post hoc* analysis was applied to compare multiple groups. Statistical analyses were performed with R (version 4.1.1) software and GraphPad Prism (version 8.00) software. *p*‐value < 0.05 was considered statistically significant.

## 3 RESULTS

### 3.1 Comprehensive analysis of human datasets

**FIGURE 2:**
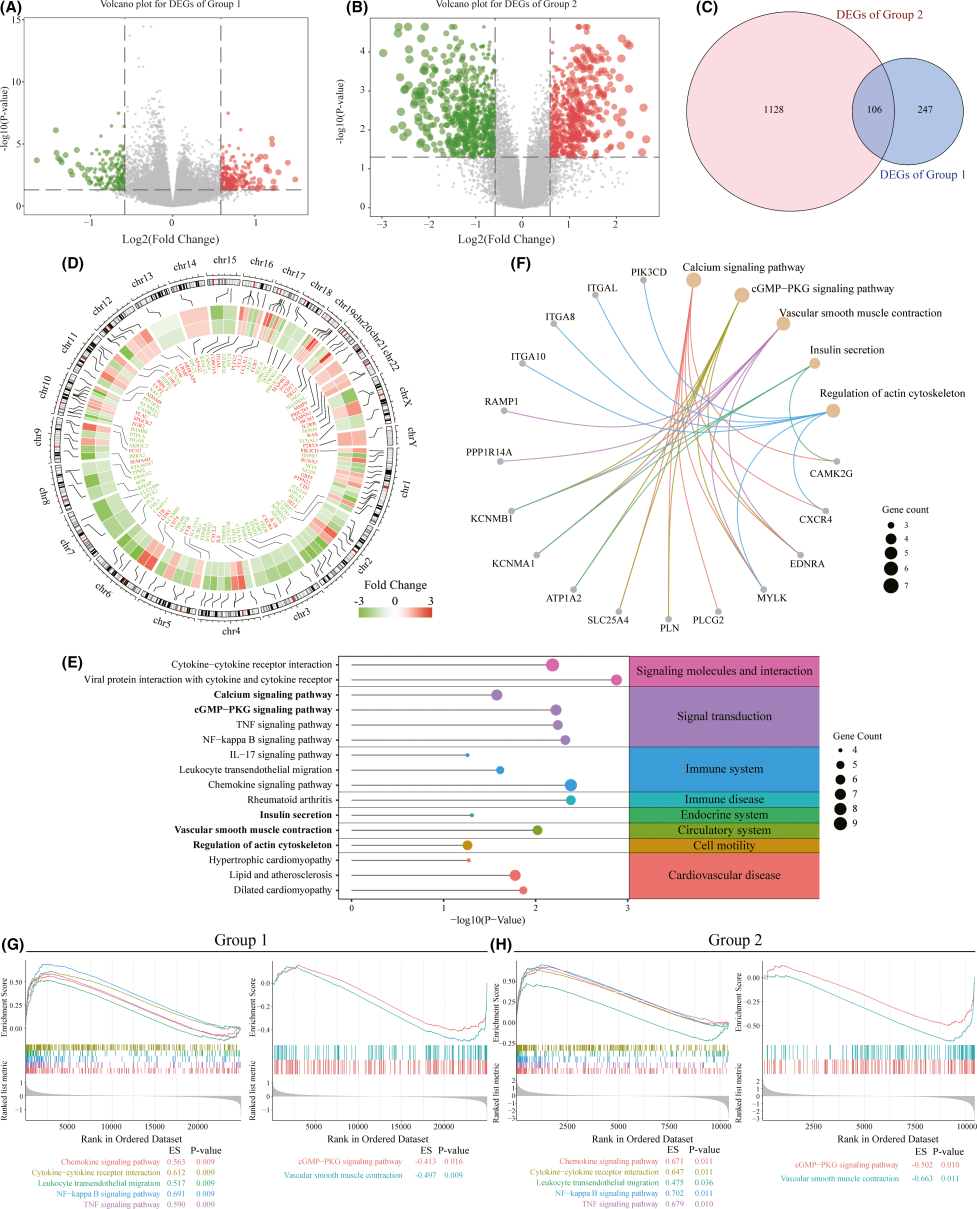
Comprehensive bioinformatics analysis of human datasets. (A, B) Volcano plots displaying the differentially expressed genes (DEGs) in Group 1 (A) and Group 2 (B) with a *p*‐value < 0.05 and fold changes >1.5. The red and green plots represent the upregulated and downregulated genes, respectively. (C) Venn diagram showing the overlap of the DEGs in Groups 1 and 2. (D) The inner heatmap of the Circos plot showing the 106 common DEGs in Groups 1 and 2. Red colours represent upregulated genes; green colours represent downregulated genes. (E) KEGG pathway enrichment analysis of the 106 common DEGs in Groups 1 and 2. The pathways with *p*‐values <0.05 are considered significant. (F) Five non‐immune pathways containing 16 common DEGs in Groups 1 and 2. (G, H) Gene set enrichment analysis (GSEA) of Group 1 (G) and Group 2 (H) showing the common KEGG pathways with adjusted *p*‐value < 0.05

After data preprocessing, the normalized data were presented in boxplots (Figure S1A and B) which showed the similar distributions between samples, indicating the reliability of the data. Based on the screening strategy with *p*‐value < 0.05 and FC >1.5, 353 DEGs, including 194 upregulated genes and 159 downregulated genes, were identified in Group 1, while 1234 DEGs, including 561 upregulated genes and 673 downregulated genes, were identified in Group 2 (Figure 2A, B). Then, we identified 106 common DEGs between Groups 1 and 2, namely 44 upregulated DEGs and 62 downregulated DEGs in human AAA samples (Figure 2C). The relative expression levels of these genes and their distribution on the chromosomes are shown in Figure 2D. In addition, we investigated the functional enrichment of these common DEGs and found that these genes were specifically enriched in the biological process (BP) of immune‐related pathways (e.g. leukocyte cell–cell adhesion, T‐cell activation and neutrophil migration). The significantly enriched cellular component (CC) terms were mainly associated with sarcomeres, contractile fibre parts and myofibrils. For molecular function (MF), the common DEGs were mainly enriched in cytokine binding, actin binding and structural constituents of muscle (**Table** **S1**). The KEGG pathway analysis revealed that the common DEGs were mainly involved in cardiovascular disease pathways, immune‐disease/‐system related pathways and signal transduction/interaction pathways (Figure 2E, F). Furthermore, GSEA also confirmed the enriched pathways in AAA, such as the chemokine signalling pathway, cytokine–cytokine receptor interaction, leukocyte transendothelial migration, the NF‐kappa B signalling pathway, the TNF signalling pathway, the cGMP‐PKG signalling pathway and vascular smooth muscle contraction **(**Figure 2G, H). These results indicated that abnormal inflammation, VSMC phenotype switching and cytokine secretion are hallmarks of human AAA. However, among the functional enrichment results, what aroused our attention is non‐immune‐related pathway, especially cGMP‐PKG signalling pathway, calcium signalling pathway, insulin secretion, vascular smooth muscle contraction and regulation of actin cytoskeleton, which contain 16 common DEGs in AAA (Figure 2F).

### 3.2 Identification of ATP1A2 as the key gene involved in AAA

**FIGURE 3:**
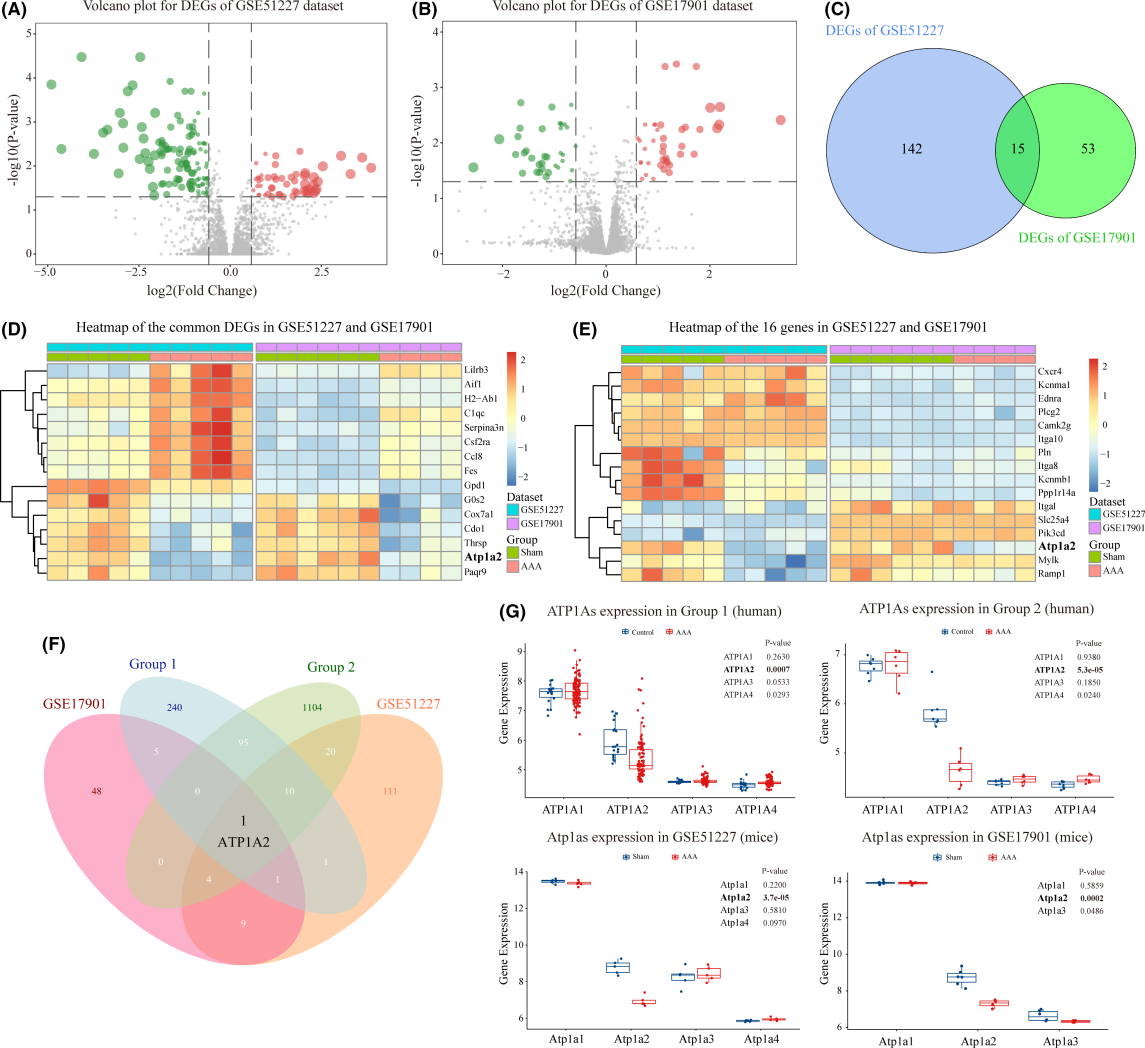
Identification of ATP1A2 in human and mouse datasets. (A, B) Volcano plots showing the differentially expressed genes (DEGs) in GSE51227 (A) and GSE17901 (B) with *p*‐value <0.05 and fold change >1.5. The red or green plots represent the upregulated or downregulated genes, respectively. (C) Venn plot displaying the fifteen common DEGs both in GSE51227 and GSE17901. (D) Heatmap demonstrating the expression profiles of the common DEGs both in GSE51227 and GSE17901. (E) Heatmap displaying the expression profiles of the 16 DEGs involved in five non‐immune pathways enriched by human datasets in GSE51227 and GSE17901. (F) Venn diagram showing ATP1A2 as the only common DEG between human and mouse datasets. (G) The expression of ATP1As in the human (Groups 1 and 2) and mouse (GSE51227 and GSE17901) datasets. ATP1A2 was significantly decreased in all datasets

To further validate the bioinformatics analysis results of the human datasets, we explored the expression profile of mouse AAA datasets (GSE51227 & GSE17901). After data preprocessing (Figure S1C and D), a total of 157 DEGs were identified in the GSE51227 dataset, including 51 upregulated and 106 downregulated genes (Figure 3A). In the GSE17901 dataset, 68 DEGs (36 upregulated and 32 downregulated genes) were identified (Figure 3B). Furthermore, we found 15 overlapping DEGs in these two datasets, which were shown in the Venn diagram and heatmap plot (Figure 3C, D). In addition, we validated the expression of 16 common DEGs involved in five non‐immune pathways (shown in Figure 2F) in mouse AAA datasets, and found a decreased expression of ATP1A2 in AAA group (Figure 3E).

Notably, ATP1A2 was the only common DEGs that was significantly downregulated in both human and mouse AAA samples after intersecting the DEGs of Groups 1–4 (Figure 3F), which suggested the ATP1A2 as a conservative regulator involved in human and mouse aneurysms. As we known, ATP1A2 belongs to the subfamily of Na^+^/K^+^‐ATPases which is composed of two subunits, alpha and beta.^31^ Four well‐known isoforms of the catalytic α‐subunit (α1, α2, α3 and α4, which are encoded by ATP1A1, ATP1A2, ATP1A3 and ATP1A4) have been identified.^32^ Given that the expression level of ATP1A2 was decreased in AAA samples, we were curious about the expression levels of other paralogs during AAA. The results showed that α1 and α2 subunits encoded by ATP1A1 and ATP1A2 genes are mainly α‐subunits of Na^+^/K^+^‐ATPases presented in the aorta (Figure 3G), which was consistent with the previous studies.^14^, ^32^, ^33^ Although ATP1A4 was significantly elevated in human AAA samples, there was no significant difference between AAA and sham samples in mice, while significant expression level of ATP1A2 rather than ATP1A1 was observed between the control and AAA groups in both human and mice (Figure 3G). Above results identified ATP1A2 as a key gene involved in the pathological condition of AAA, which reminded us whether ATP1A2 expression is cell‐specifically decreased in AAA.

### 3.3 Cellular localization of the ATP1A2 in AAA

**FIGURE 4:**
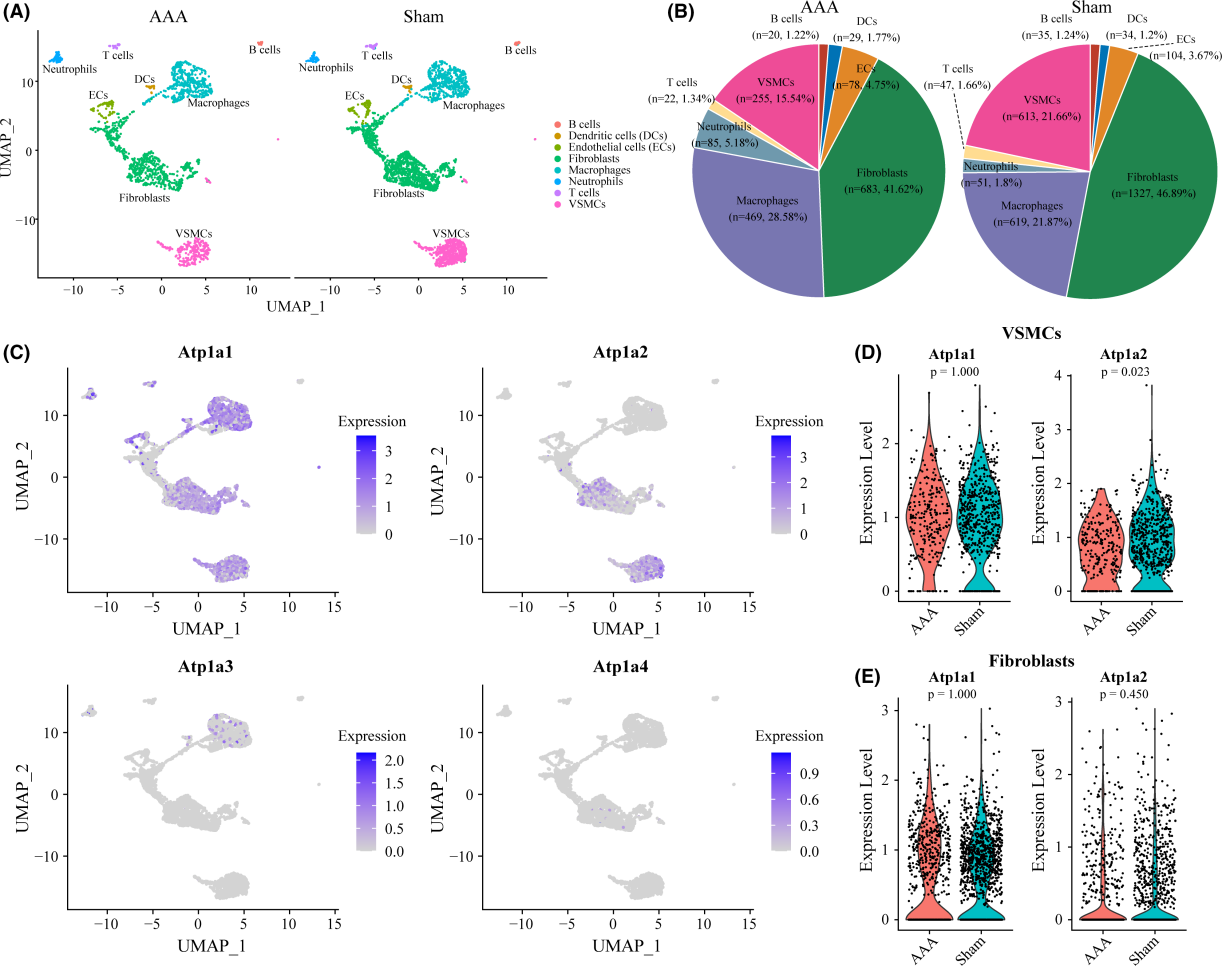
Identification of cellular localization of ATP1A2 by scRNA‐seq analysis. (A) Cell distributions in AAA and sham samples are shown in uniform manifold approximation and projection (UMAP) plot. After quality control, 1641 and 2830 cells from AAA and sham groups are captured for clustering analysis, respectively. (B) The percentages of each cell population in AAA and sham groups are exhibited in Pie plot. (C) UMAP plots display the expression levels of Atp1a1, Atp1a2, Atp1a3 and Atp1a4 in all aortic cell clusters. (D, E) Comparisons of the expression of Atp1a1 and Atp1a2 in VSMCs (D) and fibroblasts (E) between AAA and sham samples are shown in violin plots

In order to reveal the underlying mechanisms of ATP1A2 in AAA, we identified the aortic cellular localization of ATP1A2 by scRNA‐seq data analysis. After performing the individual data quality control filters, a total of 4471 cells were retrieved for subsequent analysis, including 1641 cells and 2830 cells from the AAA or sham group, respectively (Figure 4A). The CAA algorithm of Seurat package^26^ was applied to integrate the AAA and sham data, and unbiased cell clustering analysis identified 16 clusters representing eight‐cell lineages such as VSMCs, endothelial cells (ECs), fibroblasts, macrophages, neutrophils, dendritic cells (DCs), T cells and B cells (Figure S2A and S2B). Notably, the cell type‐specific markers obtained from CellMarker database^27^ were used to distinguish cell lineages (Figure S2C). Compared with the sham group, the obvious changes of cell composition in AAA were observed including decrease of VSMCs (AAA vs. Sham: 15.54% vs. 21.66%) and fibroblasts (AAA vs. Sham: 41.62% vs. 46.89%), but increase of immune cell populations (Figure 4B), which were consistent with previous study.^25^ Next, we assessed the expression levels of ATP1A1, ATP1A2, ATP1A3 and ATP1A4 in each cell population after identifying the cell type of each cell cluster (Figure 4C). Similar to the results above described, α‐subunits of Na^+^/K^+^‐ATPase presented in the aorta are mainly encoded by ATP1A1 and ATP1A2, in contrast, ATP1A3 and ATP1A4 are seldom expressed in aortic cells. In addition, unlike ATP1A1 which is ubiquitously expressed in a variety of cells, for example VSMCs, fibroblasts, macrophages, neutrophils, T cells and ECs, ATP1A2 is specifically expressed in VSMCs and fibroblasts (Figure 4C). Furthermore, when we focused on the RNA expression levels of ATP1A1 and ATP1A2 in VSMCs and fibroblasts, we found that there was no significant difference between AAA and sham samples in fibroblasts, while ATP1A2 showed a decreased expression in VSMCs (Figure 4D, E). In brief, these results suggested that among the four isoforms of the α‐subunit, ATP1A2 was significantly decreased in AAA, and its reduction mainly occurred in VSMCs of the aorta, which required further experimental verification.

### 3.4 Validation of the ATP1A2 expression in human and mouse aortic aneurysm

**FIGURE 5:**
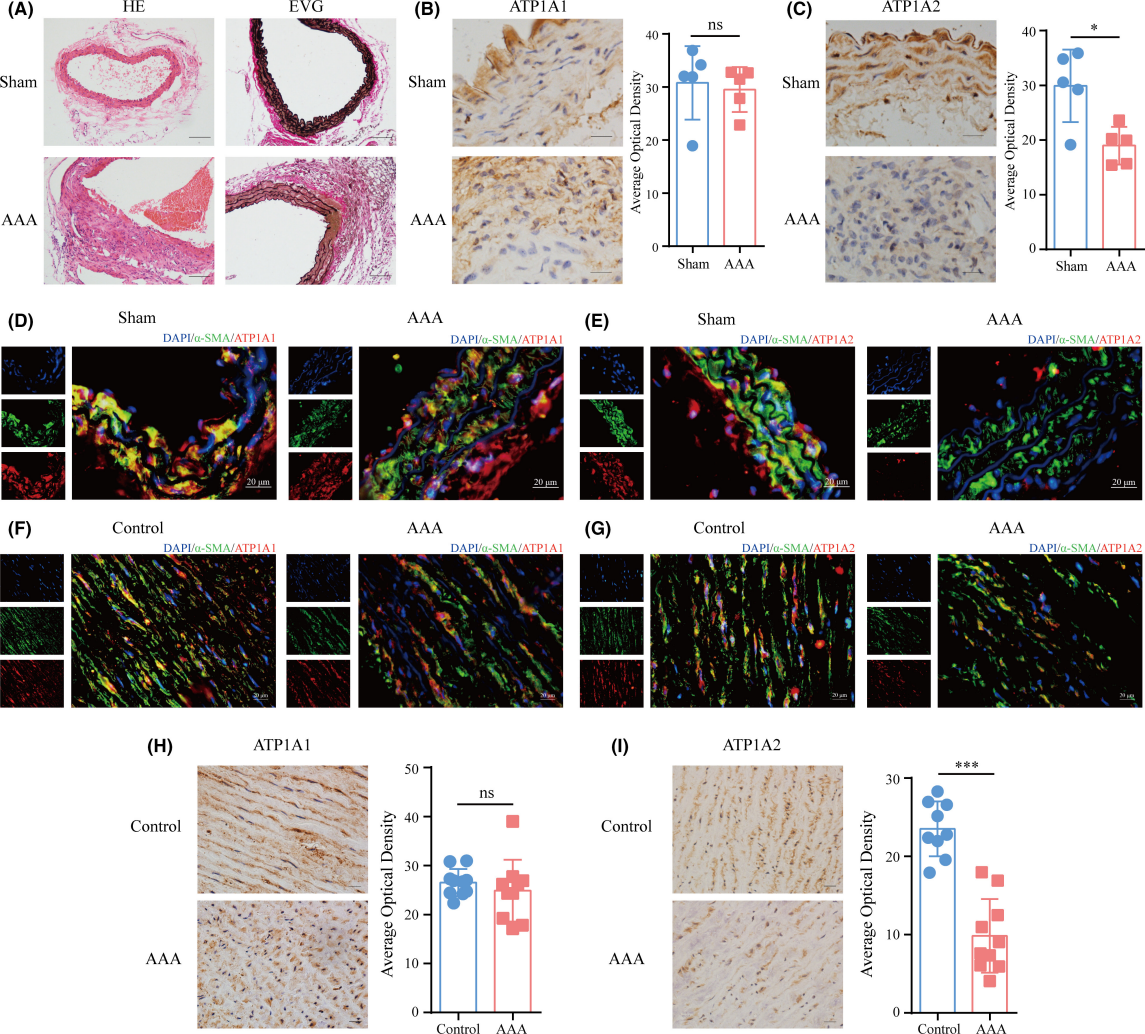
Validation of ATP1A1 and ATP1A2 expression in AAA. (A) Representative images of haematoxylin–eosin (HE) and Elastica van Gieson (EVG) staining in Ang II‐induced AAA mouse samples. Scale bar, 100 µm. (B, C) Immunohistochemical (IHC) staining shows the expression levels of ATP1A1 (B) and ATP1A2 (C) in the abdominal aorta of Sham or AAA mice. Scale bar, 20 µm. (D, E) Immunofluorescence (IF) staining for ATP1A1 (red)/α‐SMA (green) (D) or ATP1A2 (red)/α‐SMA (green) (E) in the abdominal aorta of Sham or AAA mice. Scale bar, 20 µm. (F, G) IF staining for ATP1A1 (red)/α‐SMA (green) (F) or ATP1A2 (red)/α‐SMA (green) (G) in human AAA and control samples. Scale bar, 20 µm. (H, I) IHC staining shows the expression levels of ATP1A1 (H) and ATP1A2 (I) in human AAA and control samples. Scale bar, 20 µm. Data are presented as the mean ± SD. *p < 0.05; ***p < 0.001; ns indicates no significance vs. sham/control group, *n* = 5/10, Student's *t*‐test

Since ATP1A1 and ATP1A2 are the two main isoforms expressed in the aorta,^14^ we verified the expression and distribution of ATP1A1 and ATP1A2 in human and mouse specimens by using immunohistochemistry and immunofluorescence. HE and EVG staining showed that AAA was successfully induced by Ang II infusion in ApoE^−/−^ mice (Figure 5A). Compared with saline treatment, Ang II remarkably reduced the ATP1A2 protein level in the abdominal aorta of the mice, while the expression level of ATP1A1 was similar in these two groups (Figure 5B, C). In addition, we observed extensive co‐localization of ATP1A1/2 and α‐SMA (a biomarker for VSMC) in aortic samples, demonstrating the predominant expression of ATP1A1 and ATP1A2 in VSMCs (Figure 5D, E). Furthermore, we detected the protein levels of ATP1A1 and ATP1A2 in human samples. The results showed that ATP1A1 and ATP1A2 were highly expressed in VSMCs of the normal aorta (Figure 5F, G), and the protein level of ATP1A2 was dramatically reduced in the aorta of patients with AAA (Figure 5G‐I), which confirmed the above analysis results.

Next, we were curious whether the expression patterns of ATP1A1 and ATP1A2 were limited to AAA or conserved in TAA and AD. Thus, IHC was used to detect the expression levels of ATP1A1 and ATP1A2 in the aortas of patients with or without TAA/AD. The results demonstrated that ATP1A2 was also downregulated in the aortas of patients with TAA, while there was no significant difference in ATP1A1 between the two groups (Figure S3A and S3B). The same expression pattern (i.e. significant reduction in ATP1A2 expression) was also observed in AD samples (Figure S3C and S3D). Moreover, the Western blot results further confirmed the decrease in ATP1A2 expression in AAA and TAA samples (Figure S3E). Thus, these results indicated that ATP1A2 may be a key regulator of both AAA and TAA.

### 3.5 Identification of potential TFs regulating ATP1A2 expression

**FIGURE 6:**
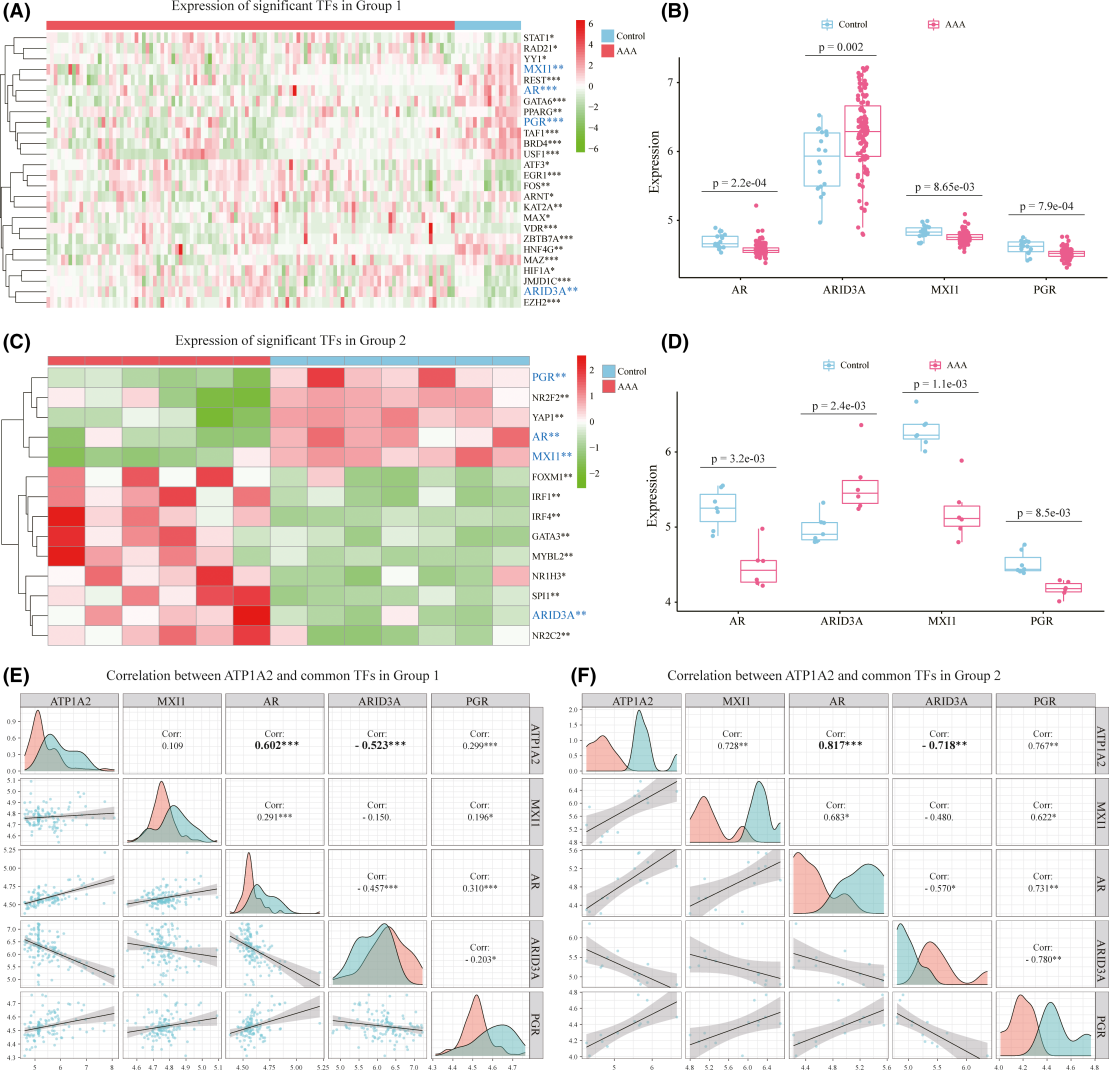
Correlation analysis of ATP1A2 and potential TFs. (A, C) Heatmaps show the significantly expressed TFs that target ATP1A2 in Group 1 (A) and Group 2 (C), which are filtered by the Signaling Pathways Project database. (B, D) The expression levels of 4 common TFs (AR, ARID3A, MXI1 and PGR) in Group 1 (B) and Group 2 (D) are shown in boxplots. (E, F) Correlation analysis of ATP1A2 and four common TFs in Group 1 (E) and Group 2 (F)

The GEO data and our own verification data both show that the expression level of ATP1A2 is significantly reduced in aneurysms. Therefore, it is very interesting to reveal the molecular mechanism that regulates the reduction in ATP1A2 expression. TFs are one of the most important molecules that regulate gene expression by directly binding to the promoters of target genes.^34^ Thus, to identify the TFs that regulate the expression of ATP1A2, the Signaling Pathways Project database is used to predict the TFs that bind to the promoter of ATP1A2.^28^ A total of 103 TFs were obtained under the limit of ‘human’ in the Biosample Category and FDR <0.05. Then, we further screened these 103 TFs in Group 1 and Group 2 with adjusted *p*‐values <0.05. Twenty‐six TFs in Group 1 and fourteen TFs in Group 2 were eligible (Figure 6A, C). Among them, four TFs (AR, ARID3A, MXI1 and PGR) had the same expression pattern in Groups 1 and 2 (Figure 6B, D). To identify the TFs that potentially regulate ATP1A2, we performed Pearson's correlation analysis of ATP1A2 and common TFs (AR, ARID3A, MXI1 and PGR) in Groups 1 and 2 (Figure 6E, F). By applying absolute value of correlation coefficients >0.5 and *p*‐values <0.05, AR and ARID3A were shown to be correlated with ATP1A2 in both Group 1 and Group 2; specifically, AR was positively correlated with ATP1A2, and ARID3A was negatively correlated with ATP1A2 in the two groups (Figure 6E, F).

### 3.6 ARID3A negatively regulates ATP1A2 expression in VSMCs

**FIGURE 7:**
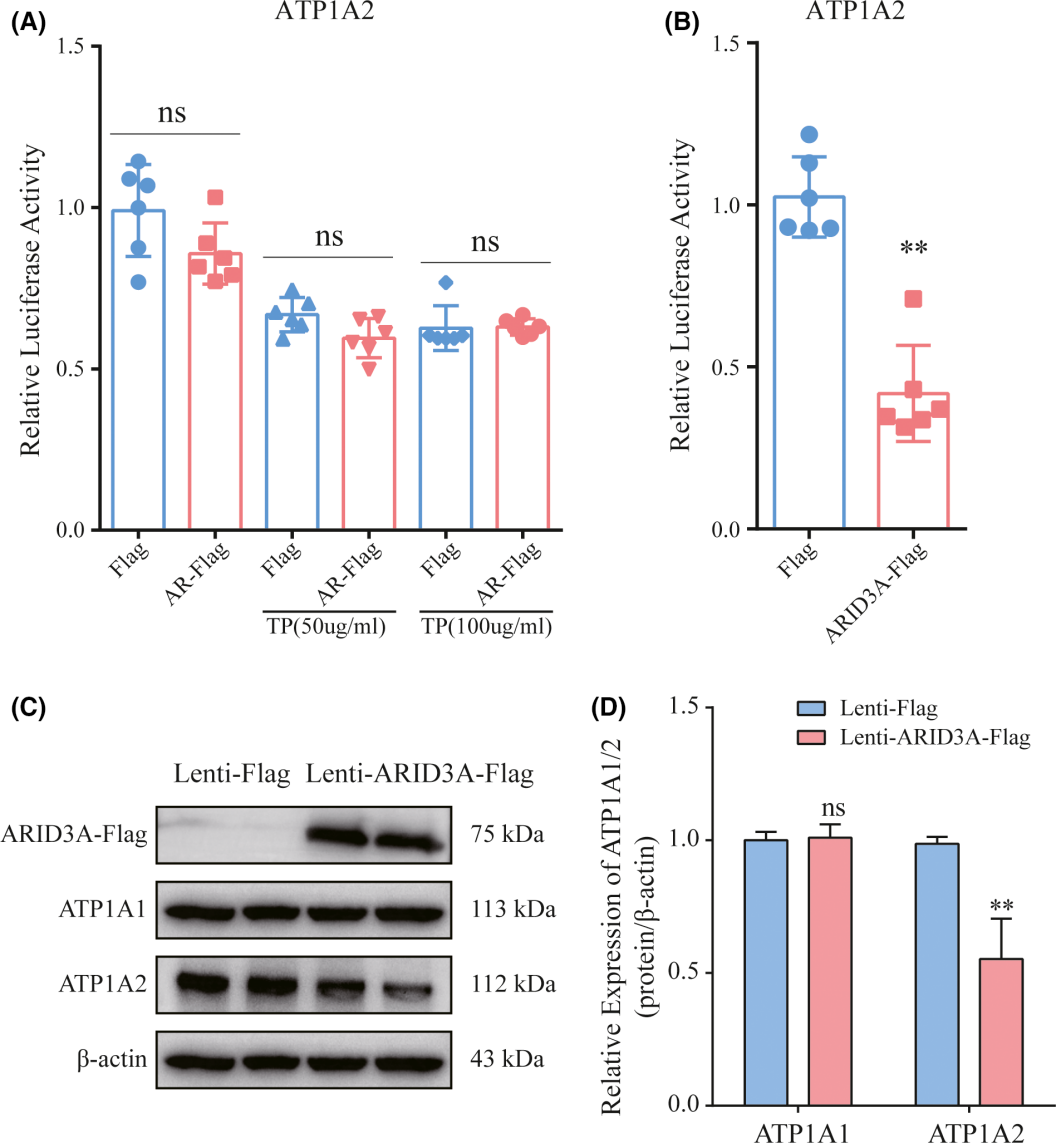
ARID3A inhibits the expression of ATP1A2. (A) Luciferase assays showing the effects of AR overexpression on the regulation of ATP1A2 expression (*n* = 6), TP: testosterone propionate. (B) Luciferase assays showing the effects of ARID3A overexpression on the regulation of ATP1A2 expression (*n* = 6). (C, D) Overexpression of ARID3A decreased the expression level of ATP1A2, as determined by Western blot (*n* = 3). Data are presented as the mean ± SD. **p < 0.01; ns indicates no significance vs. controls, Student's *t*‐test.

Based on the correlation analysis of ATP1A2 and candidate upstream TFs, AR and ARID3A were regarded as potential TFs that regulate ATP1A2. Then, we cotransfected HEK293T cells overexpressing AR or ARID3A plus an ATP1A2 luciferase reporter vector. Given that AR requires activation by androgen to play a role in transcriptional regulation,^35^ we adopted different concentrations of androgen analogs (testosterone propionate, TP) to activate AR. The results showed that regardless of TP stimulation, overexpression of AR did not significantly increase the expression of ATP1A2 (Figure 7A), while overexpression of ARID3A resulted in an approximately 50% reduction in ATP1A2 (Figure 7B). Furthermore, ARID3A was overexpressed in primary cultured human aorta smooth muscle cells (HASMCs) through lentivirus, and ATP1A2, but not ATP1A1 was obviously suppressed by ARID3A overexpression (Figure 7C, D). These results indicate that ARID3A is the TF that binds to the promoter of ATP1A2 and inhibits its expression. Furthermore, scRNA‐seq data analysis implies that the increased ARID3A in AAA might be derived from macrophages (Figure S4).

## 4 DISCUSSION

Although the underlying pathophysiological processes responsible for AAA have been extensively studied in recent decades, the mechanisms associated with these processes during the formation and progression of AAA are still incompletely understood. To provide novel insights into the pathological mechanism of AAA, the present study utilized human and mouse datasets from the GEO database to assess the transcriptome profiles of aneurysmal and nonaneurysmal abdominal aorta. Furthermore, functional enrichment analysis was performed to provide a greater understanding of the processes involved in AAA, which provided us with a more global picture of the pathology based on the annotated pathways rather than isolated genes. In addition, the current study identified a new candidate gene, ATP1A2, which was downregulated in all datasets and further verified in experimental studies. We believe that reduction in ATP1A2 contributes to the initiation and progression of AAA, affecting vascular integrity due to its roles in the regulation of blood pressure and ion homeostasis.

As we currently known, ATP1A2 gene encodes an α2‐subunit of Na^+^/K^+^‐ATPase which is firstly discovered as the molecular machine for pumping Na^+^ and K^+^ across cell membrane.^36^ Na^+^/K^+^‐ATPase is composed of two essential subunits, alpha (the catalytic subunit of the enzyme) and beta. There are four isoforms of the α‐subunit of Na^+^/K^+^‐ATPase such as α1, α2, α3 and α4.^13^, ^31^ Among them, the α1 and α2 subunits encoded by ATP1A1 and ATP1A2 genes are present in the aorta (70% of all Na^+^/K^+^‐ATPases in mice contain the α1‐subunit, and the remaining 30% contain the α2‐subunit),^14^, ^32^, ^33^ which is similar to our results. Although α2‐subunit exhibits low expression in the cardiovascular system compared with the α1‐subunit, previous studies have confirmed its regulation for cardiovascular function and blood pressure by applying a global genetic knockout of α2‐subunit mice.^37^, ^38^ Currently, no study has revealed the relationship between Na^+^/K^+^‐ATPase and AAA, but some studies have found its roles in regulating the contraction of VSMCs, thereby leading to dynamic changes in blood pressure.^14^, ^39^

Currently, the regulatory effects of Na^+^/K^+^‐ATPase on VSMCs are mainly manifested in the following three aspects:

1. Na^+^/K^+^‐ATPase maintains intracellular ion homeostasis.
  It is well known that Na^+^/K^+^‐ATPase maintains high cytosolic K^+^ and low cytosolic Na+concentrations in animal cells by transporting two K^+^ ions into the cell and three Na^+^ ions out of the cell with the hydrolysis of an ATP molecule,^40^ which is essential for maintaining the resting membrane potential and excitability. Many cellular processes and functions depend on the electrochemical gradient established by Na^+^/K^+^‐ATPase, for instance, transport of glucose and amino acids into the cell, excitability of muscle and nerves, and regulation of the intracellular concentrations of other ions including Ca.^2^ ^+^ ^38^, ^41^ Compared with homogeneous distribution of α1‐isoform in VSMCs, α2‐isoform is localized in restricted submembranous areas closed to the Na^+^, Ca^2+^‐exchanger (NCX), which enables to influence the local Na^+^/Ca^2+^ concentration via NCX.^42^ As Shelly et al. reported, the ionic current generated by the α2‐isoform is recorded smaller than expected in relation to their expression ratio in VSMCs (α1: α2 = approximately 7: 3), which implies that the α2‐isoform is less active at resting conditions but may be activated during abnormal agonist stimulation.^32^, ^43^
2. Na+/K+‐ATPase is involved in VSMC apoptosis and proliferation.
  It has been reported that inhibition of Na^+^/K^+^‐ATPase suppresses apoptosis of VSMCs in various unfavourable conditions. For example, Na^+^/K^+^‐ATPase inhibition by ouabain (a special type of Na^+^/K^+^‐ATPase inhibitor) sharply suppressed apoptosis in serum‐deprived VSMCs.^44^ Knocking out ATP1A2 suppressed the rate of apoptosis following PM2.5 exposure.^45^ The apoptosis of VSMCs and cardiomyocytes transfected with ATP1A2 siRNA was alleviated in anoxia/reoxygenation injury.^46^ Notably, in addition to the apoptosis, some studies also demonstrated that Na^+^/K^+^‐ATPase was involved in VSMC proliferation. A novel butyrolactone derivative could inhibit migration and proliferation of VSMCs through the inhibition effects on the activity of Na^+^/K^+^‐ATPase.^47^ Furthermore, low concentrations of ouabain reflecting the affinity to the α1‐isoform can activate proliferation via Src/EGFR and ERK1/2 activation in the synthetic phenotype of VSMCs.^48^ The Ang II, a molecule used to induce AAA mouse model, could stimulate Na^+^/K^+^‐ATPase activation by upregulating ATP1A1/2 gene transcription via PI3K‐p42/44 signal pathways, which was responsible for Ang II‐induced VSMCs proliferation.^49^ The above findings confirmed the crucial roles of Na^+^/K^+^‐ATPase in determining the fate of VSMCs during the pathological conditions.
3. Na+/K+‐ATPase modulates vascular tone.
  The Na^+^/K^+^‐ATPase (specifically the α2‐isoform) plays an important role in modulating VSMC tone and blood pressure.^50^ It has been elucidated that Na^+^/K^+^‐ATPase activation in VSMCs leads to membrane hyperpolarization which consequently reduces the levels of intracellular Ca^2+^, thereby contributing to vasodilation.^14^ On the contrary, the reduction of Na^+^/K^+^‐ATPase either by pharmacological inhibition or by knocking it down results in the elevated contraction of VSMCs by influencing intracellular Ca^2+^ concentrations through NCX.^14^ Furthermore, Pritchard et al. found that expressions of α‐isoforms are negatively correlated with blood pressure, and the decreased blood pressure depends on the degree of increased vascular Na^+^/K^+^‐ATPases expression.^51^ Especially, the α2‐isoform is most important for the regulation of blood pressure based on the previous studies. It is reported that α1‐isoform heterozygote knockout mice have normal blood pressure.^52^ In contrast, heterozygote mice with global α2‐isoform knockout and mice with VSMC‐specific dominant‐negative α2‐isoform expression have elevated blood pressure, while mice overexpressing the α2‐isoform are hypotensive.^53^

Hypertension has been considered as a potential risk factor for AAA. Epidemiological studies report that blood pressure and cholesterol control and smoking cessation could reduce the number of patients that develop AAA.^54^ At present, elevated blood pressure is considered to be mediated by activation of the renin–angiotensin system (RAS) and upregulation of Ang II.^2^, ^10^ A well‐established AAA mouse model that employs long‐term Ang II infusion recapitulates many aspects of human AAA, including elevated blood pressure, dependence on higher Ang II levels and gradual recruitment of immune cells.^2^ Our study reported that the ATP1A2 specifically expressed in VSMCs was significantly downregulated in AAA, which was confirmed to have the ability to regulate blood pressure in many previous studies, thus implying a potential therapeutic target for patients with hypertension and AAA.

To determine the mechanisms responsible for ATP1A2 downregulation, we focused on the TFs that target ATP1A2. A total of 103 potential TFs were predicted by the Signaling Pathways Project database. Among them, only four TFs (AR, ARID3A, MXI1 and PGR) were significantly differentially expressed in the human datasets. Finally, through correlation analysis and experimental verification, we found that ARID3A negatively regulated the expression of ATP1A2. ARID3A, also known as Bright, was originally discovered for promoting immunoglobulin transcription in antigen‐activated B cells.^55^ As a transcription factor, it interacts with DNA through its A/T‐rich interacting domain, which has been confirmed to have epigenetic regulatory functions.^56^ In addition, the expression of ATP1A2 is reported to be regulated by TFs. Wales et al.^57^ identified ATP1A2 as the MEF2A target gene by ChIP‐seq analysis in differentiating myoblast cells and cardiomyocytes, and demonstrated that loss of MEF2A resulted in the downregulation of APT1A2. Regarding the potential target genes regulated by ARID3A, the results of two ChIP‐seq datasets examining ARID3A knockout or knock‐down in K562 cells revealed 1728 dysregulated DEGs and 1065 dysregulated DEGs, respectively.^58^ Among the DEGs, ATP1A2 was upregulated in both datasets, implicating the negative regulatory effect of ARID3A on ATP1A2, which was consistent with our results.

Currently, various studies have used the scRNA‐seq technique to delineate the heterogeneity of vascular cells in aortic aneurysm (AAA and TAA). Yang et al., Zhao et al. and Li et al.^25^, ^59^, ^60^ performed scRNA‐seq on different mouse AAA models including modified CaCl2‐induced model, elastase‐induced model and Ang II‐induced model. After integrative analysis of the scRNA‐seq data, they identified multiple cell types or subtypes of AAA tissue including no‐immune cells (VSMCs, endothelial cells and fibroblasts) and immune cells (macrophages, B cells, T cells, NK cells and neutrophils). Based on the results from scRNA‐seq analysis, some novel potential mechanisms of AAA initiation and progression have been proposed. For example, Li et al.^60^ defined CD45^+^ COL1^+^ fibrocytes in AAA tissue, and the fibrocyte treatment exhibited a protective effect against AAA development. Qian et al.^61^ focused on the VSMC clusters identified by scRNA‐seq analysis and captured numbers of transcriptome with differential expression patterns in AAA. In addition, Davis et al.^62^ performed scRNA‐seq analysis on a cohort of AAA patients undergoing abdominal aortic surgery. They focused on the immune landscape in the aortic tissue and identified increased JMJD3 in aortic monocytes/macrophages resulting in upregulation of an inflammatory immune response. Furthermore, there were three groups that adopted scRNA‐seq analysis to uncover the cellular and molecular landscape of human TAA tissues with or without Marfan syndrome.^63^, ^64^, ^65^ Pedroza et al.^64^ characterized the dynamic VSMC phenotype modulation, which promoted extracellular matrix substrate modulation and aortic aneurysm progression. Among the subtypes of VSMCs, a disease‐specific signature of modulated VSMCs was identified, which might be driven by TGF‐β signalling and Klf4 overexpression. With the advance of the scRNA‐seq technology, it provides an opportunity for us to reveal the cellular heterogeneity and the gene expression profiles of AAA at single‐cell level, which will greatly facilitate the development of targeted drugs for target cells.

## 5 CONCLUSIONS

In conclusion, in the present study, we reanalysed seven AAA datasets to examine the global gene expression profile of AAA and identified ATP1A2 as a key gene involved in the progression of AAA. Furthermore, we screened TFs that potentially regulate ATP1A2, which showed that ARID3A negatively regulated ATP1A2. Considering our findings, we believe that ATP1A2 and/or ARID3A play important roles in AAA initiation and progression, which broadens our understanding of the pathogenesis of AAA, and provides valuable insights for the future AAA therapeutic research.

## CONFLICTS OF INTERESTS

The authors declare that they have no competing interests.

## AUTHOR CONTRIBUTIONS

**Qunhui Wang:** Data curation (equal); Formal analysis (equal); Visualization (equal); Writing – original draft (equal). **Na Li:** Data curation (equal); Formal analysis (equal); Investigation (equal). **Xian Guo:** Methodology (equal); Validation (equal). **Bo Huo:** Investigation (equal); Validation (equal). **Rui Li:** Data curation (equal). **Xin Feng:** Validation (equal). **Zemin Fang:** Resources (equal). **Xue‐Hai Zhu:** Data curation (equal). **Yixiang Wang:** Visualization (equal). **Xin Yi:** Data curation (equal). **Xiang Wei:** Conceptualization (equal); Project administration (equal); Supervision (equal). **Ding‐Sheng Jiang:** Conceptualization (equal); Supervision (equal); Writing – review & editing (equal).

## Supporting information

Fig S1

Fig S2

Fig S3

Fig S4

Table S1

## Data Availability

The datasets generated and analysed during the present study are included in the article/supplementary material or GEO database (http://www.ncbi.nlm.nih.gov/geo/).
