#  Corrigendum

**DOI:** 10.1111/jcmm.17484

**Published:** 2022-09-05

In Qunhui Wang et al.,^1^ the Western blot of ATP1A1 in Figure 7C is misplaced as the β‐actin, which needs to be corrected. The correct figure is shown below. The authors confirm all results, and conclusions of this article remain unchanged.

**FIGURE 7:**
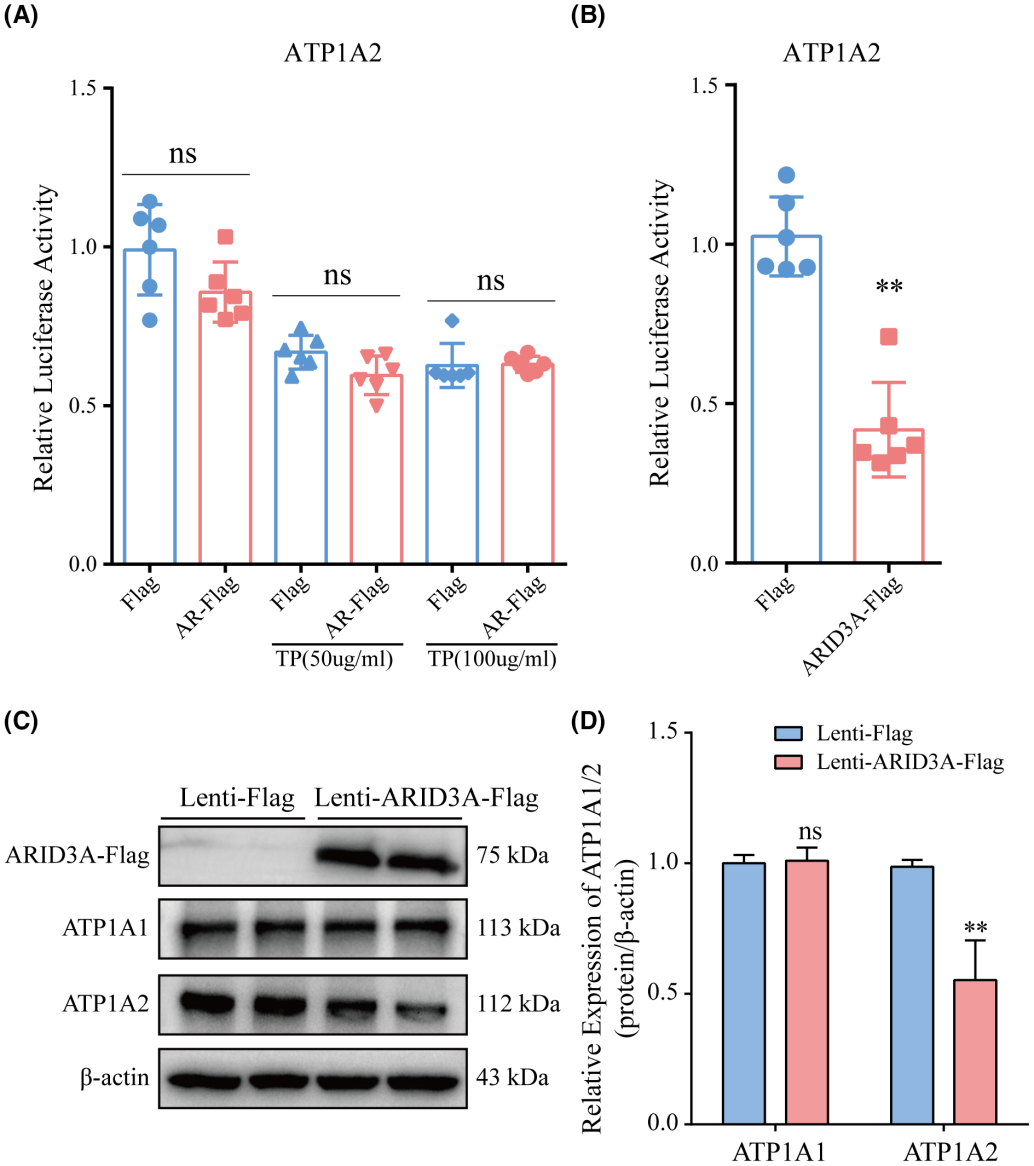
ARID3A inhibits the expression of ATP1A2. (A) Luciferase assays showing the effects of AR overexpression on the regulation of ATP1A2 expression (*n* = 6), TP: testosterone propionate. (B) Luciferase assays showing the effects of ARID3A overexpression on the regulation of ATP1A2 expression (*n* = 6). (C, D) Overexpression of ARID3A decreased the expression level of ATP1A2, as determined by Western blot (*n* = 3). Data are presented as the mean ± SD. **p < 0.01; ns indicates no significance vs. controls, Student's *t*‐test
