# Supplementary figures and images for: Comprehensive analysis identified a reduction in ATP1A2 mediated by ARID3A in abdominal aortic aneurysm
Source: J Cell Mol Med. 2022 Apr 19;26(10):2866–80. doi: 10.1111/jcmm.17301 (PMC9097831; doi:10.1111/jcmm.17301)

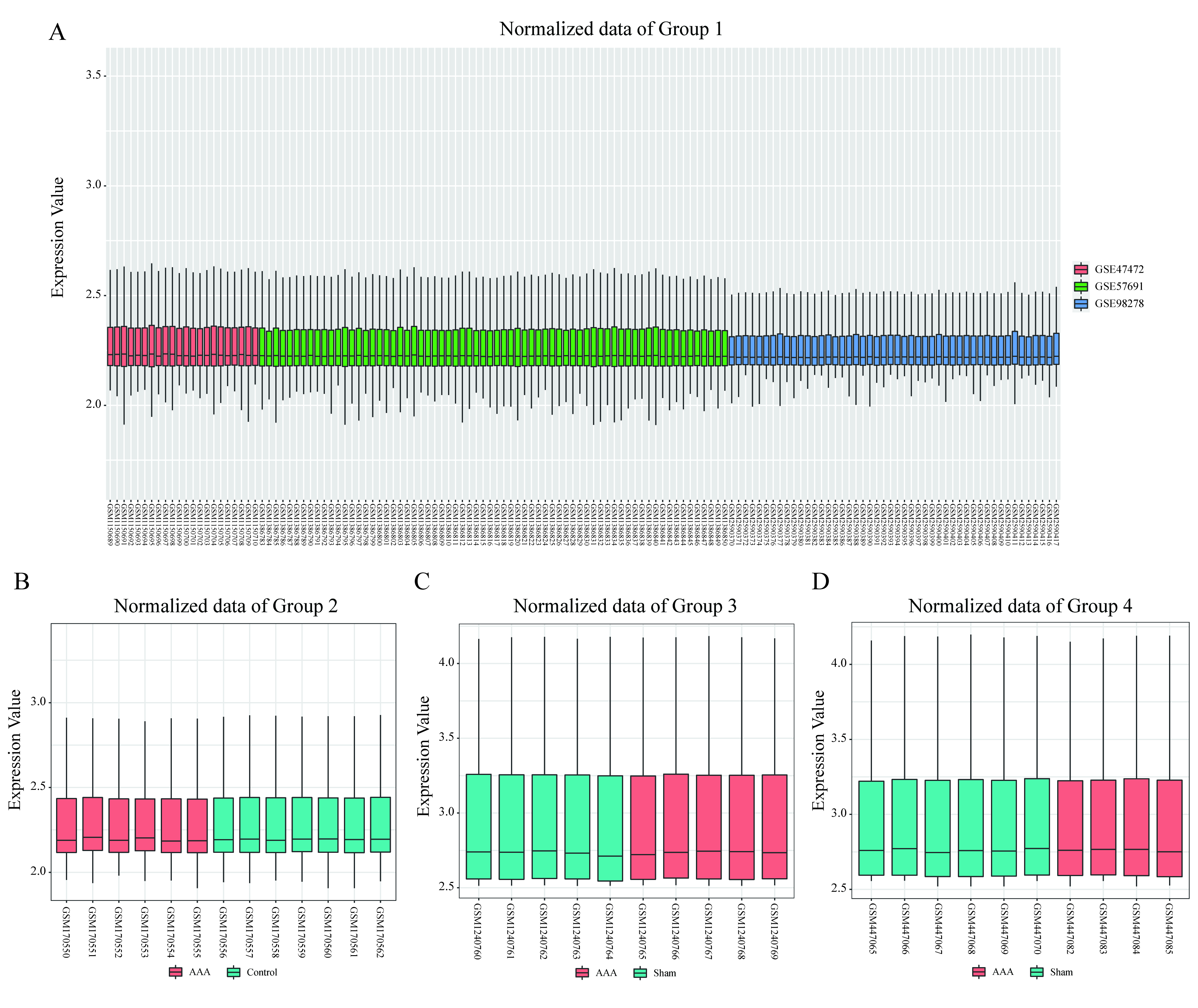

Supplement: Supplementary file 1 — Fig S1 [file JCMM-26-2866-s002.tif]

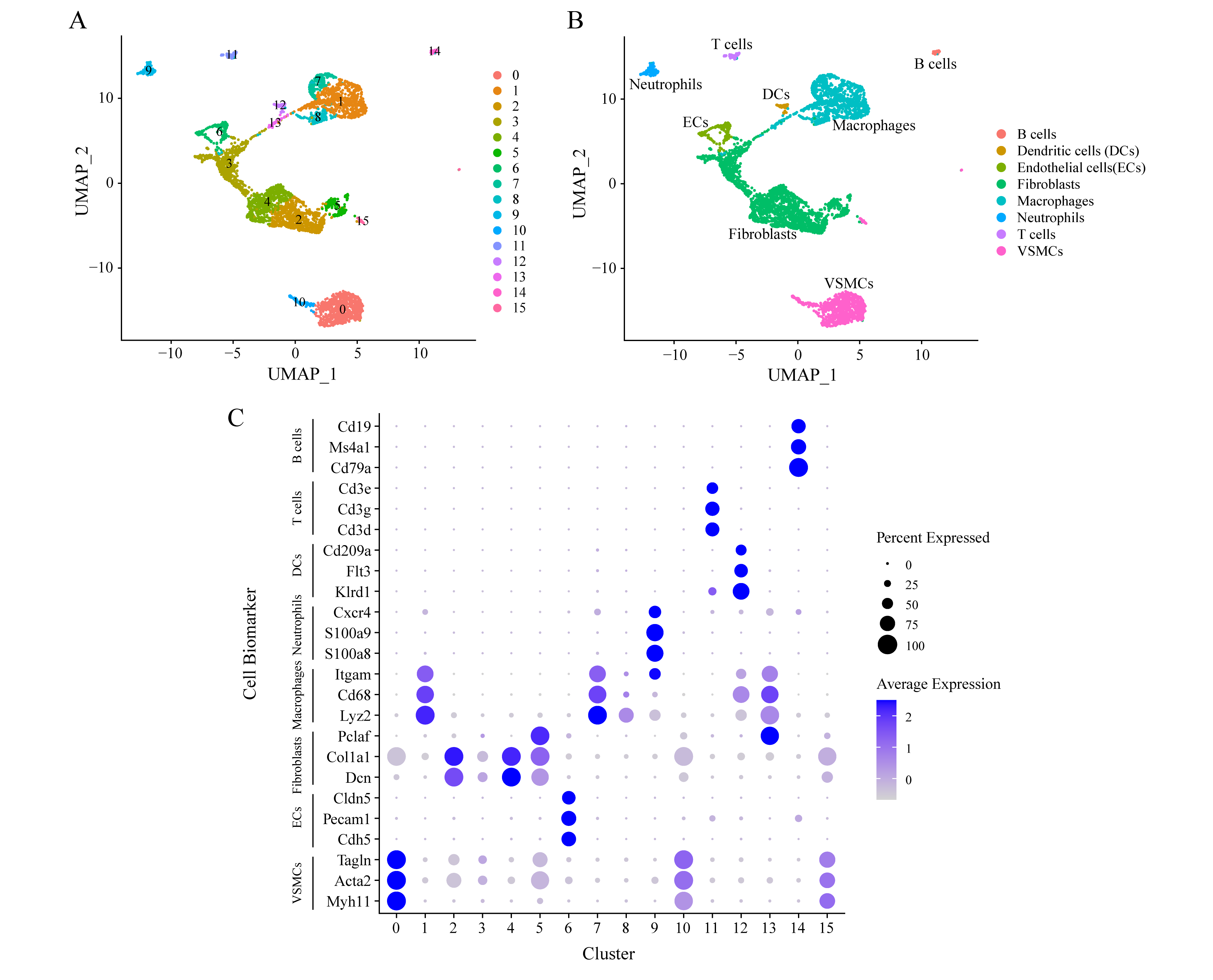

Supplement: Supplementary file 2 — Fig S2 [file JCMM-26-2866-s001.tif]

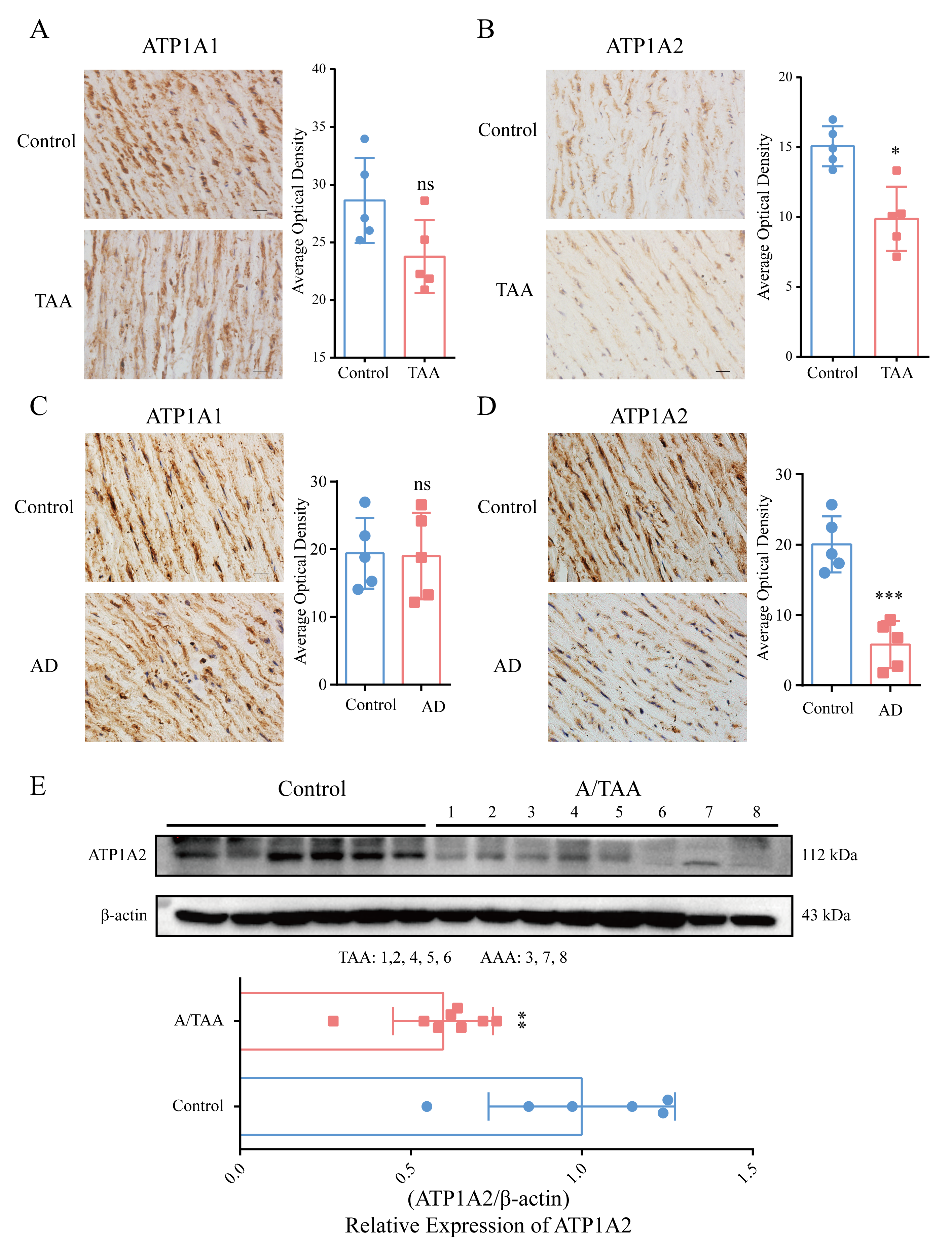

Supplement: Supplementary file 3 — Fig S3 [file JCMM-26-2866-s003.tif]

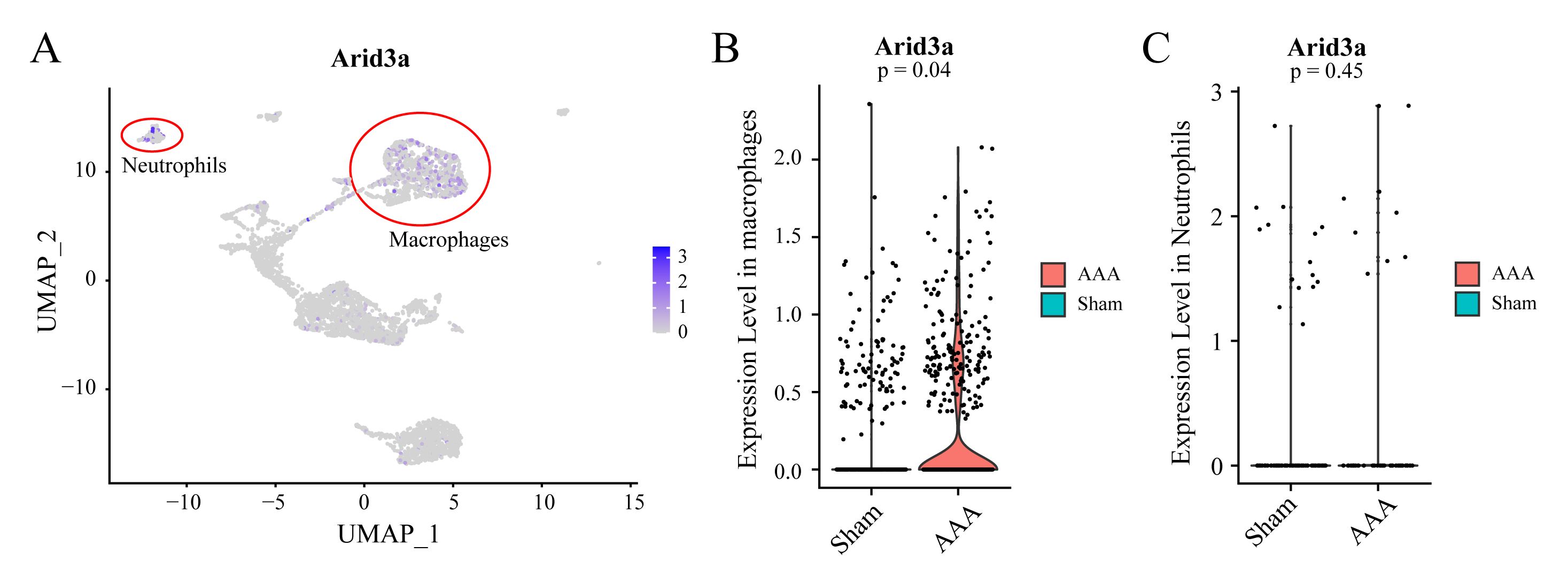

Supplement: Supplementary file 4 — Fig S4 [file JCMM-26-2866-s004.tif]
