# Supplementary material for: Comprehensive analysis identified a reduction in ATP1A2 mediated by ARID3A in abdominal aortic aneurysm
Source: J Cell Mol Med. 2022 Apr 19;26(10):2866–80. doi: 10.1111/jcmm.17301 (PMC9097831; doi:10.1111/jcmm.17301)
Supplement: Supplementary file 5 — Table S1 [file JCMM-26-2866-s005.docx]

**Supplementary Table 1**. The top 30 GO terms enriched by common DEGs of Group 1&2

| Term | Description | P-value | q-Value | Gene Symbol | Count | Group |
| --- | --- | --- | --- | --- | --- | --- |
| GO:0007159 | leukocyte cell-cell adhesion | 2.65E-05 | 1.86E-05 | ADAM8/CCR7/CD74/CD83/CORO1A/IGFBP2/IL1B/ITGAL/PTPN22/RUNX3/SELL/SEMA4D/TWSG1 | 13 | BP |
| GO:0042110 | T cell activation | 7.57E-05 | 5.30E-05 | ADAM8/CCR7/CD2/CD74/CD83/CORO1A/IGFBP2/IL1B/ITGAL/PIK3CD/PTPN22/RUNX3/TWSG1/WAS | 14 | BP |
| GO:1990266 | neutrophil migration | 9.65E-05 | 6.76E-05 | ADAM8/CCL3/CCL3L1/CCR7/CD74/CXCL2/IL1B/PIK3CD | 8 | BP |
| GO:0030595 | leukocyte chemotaxis | 9.65E-05 | 6.76E-05 | ADAM8/CCL3/CCL3L1/CCR7/CD74/CORO1A/CXCL2/CXCR4/IL1B/PIK3CD | 10 | BP |
| GO:0050863 | regulation of T cell activation | 1.69E-04 | 1.18E-04 | ADAM8/CCR7/CD2/CD74/CD83/CORO1A/IGFBP2/IL1B/PTPN22/RUNX3/TWSG1 | 11 | BP |
| GO:0001819 | positive regulation of cytokine production | 1.69E-04 | 1.18E-04 | ADAM8/BIRC3/CCL3/CCR7/CD2/CD74/CD83/FCN1/GBP5/IL1B/LTB/PLCG2/PTPN22 | 13 | BP |
| GO:1903037 | regulation of leukocyte cell-cell adhesion | 5.22E-04 | 3.66E-04 | ADAM8/CCR7/CD74/CD83/CORO1A/IGFBP2/IL1B/PTPN22/RUNX3/TWSG1 | 10 | BP |
| GO:0003012 | muscle system process | 5.68E-04 | 3.98E-04 | ATP1A2/CAMK2G/CXCR4/EDNRA/IL1B/KCNA5/KCNMA1/MYLK/MYOM1/PLN/SGCA/TPM2 | 12 | BP |
| GO:0051249 | regulation of lymphocyte activation | 7.73E-04 | 5.41E-04 | ADAM8/CCR7/CD2/CD74/CD83/CORO1A/IGFBP2/IL1B/PTPN22/RUNX3/SAMSN1/TWSG1 | 12 | BP |
| GO:0051480 | regulation of cytosolic calcium ion concentration | 1.44E-03 | 1.01E-03 | ATP1A2/CCL3/CCR7/CORO1A/CXCR4/EDNRA/KCNA5/P2RY8/PLCG2/PLN | 10 | BP |
| GO:0010959 | regulation of metal ion transport | 2.44E-03 | 1.71E-03 | ATP1A2/CAMK2G/CCL3/CORO1A/CXCR4/KCNA5/KCNMB1/MYLK/PLCG2/PLN | 10 | BP |
| GO:0030017 | sarcomere | 1.14E-04 | 7.93E-05 | CSRP1/FBXO32/FHL5/KCNA5/MYOM1/NEXN/SYNPO2/TIMP4/TPM2 | 9 | CC |
| GO:0044449 | contractile fiber part | 1.14E-04 | 7.93E-05 | CSRP1/FBXO32/FHL5/KCNA5/MYOM1/NEXN/SYNPO2/TIMP4/TPM2 | 9 | CC |
| GO:0030016 | myofibril | 1.14E-04 | 7.93E-05 | CSRP1/FBXO32/FHL5/KCNA5/MYOM1/NEXN/SYNPO2/TIMP4/TPM2 | 9 | CC |
| GO:0008305 | integrin complex | 1.59E-02 | 1.11E-02 | ITGA10/ITGA8/ITGAL | 3 | CC |
| GO:0009897 | external side of plasma membrane | 4.93E-02 | 3.44E-02 | CCR7/CD2/CD74/CD83/CXCR4/FCN1/IL2RB | 7 | CC |
| GO:0005925 | focal adhesion | 4.93E-02 | 3.44E-02 | CSPG4/CSRP1/ITGA8/NEXN/PLAU/SYNPO2/TGFB1I1 | 7 | CC |
| GO:0005924 | cell-substrate adherens junction | 4.93E-02 | 3.44E-02 | CSPG4/CSRP1/ITGA8/NEXN/PLAU/SYNPO2/TGFB1I1 | 7 | CC |
| GO:0030055 | cell-substrate junction | 4.93E-02 | 3.44E-02 | CSPG4/CSRP1/ITGA8/NEXN/PLAU/SYNPO2/TGFB1I1 | 7 | CC |
| GO:0045121 | membrane raft | 4.93E-02 | 3.44E-02 | ATP1A2/BIRC3/CD2/KCNA5/KCNMA1/SGCA | 6 | CC |
| GO:0098857 | membrane microdomain | 4.93E-02 | 3.44E-02 | ATP1A2/BIRC3/CD2/KCNA5/KCNMA1/SGCA | 6 | CC |
| GO:0098533 | ATPase dependent transmembrane transport complex | 4.93E-02 | 3.44E-02 | ATP1A2/PLN | 2 | CC |
| GO:0019955 | cytokine binding | 9.86E-03 | 6.81E-03 | CCR7/CD74/CXCR4/IL10RA/IL2RB/TWSG1 | 6 | MF |
| GO:0003779 | actin binding | 9.86E-03 | 6.81E-03 | CORO1A/CXCR4/KCNMA1/MAP1B/MYLK/MYOM1/NEXN/SYNPO2/TPM2/WAS | 10 | MF |
| GO:0008307 | structural constituent of muscle | 9.86E-03 | 6.81E-03 | CSRP1/MYOM1/NEXN/TPM2 | 4 | MF |
| GO:0042805 | actinin binding | 9.86E-03 | 6.81E-03 | CSRP1/KCNA5/MYOM1/SYNPO2 | 4 | MF |
| GO:0004896 | cytokine receptor activity | 1.22E-02 | 8.41E-03 | CCR7/CD74/CXCR4/IL10RA/IL2RB | 5 | MF |
| GO:0008131 | primary amine oxidase activity | 2.43E-02 | 1.68E-02 | AOC3/MAOA | 2 | MF |
| GO:0005518 | collagen binding | 2.43E-02 | 1.68E-02 | ITGA10/MMP9/PCOLCE2/SPOCK2 | 4 | MF |
| GO:0019838 | growth factor binding | 3.92E-02 | 2.71E-02 | FGFRL1/IGFBP2/IL10RA/IL2RB/TWSG1 | 5 | MF |
| BP: biological process; CC: cellular component; MF: molecular function | | | | | | |
